# Cancers Screening in an Asymptomatic Population by Using Multiple Tumour Markers

**DOI:** 10.1371/journal.pone.0158285

**Published:** 2016-06-29

**Authors:** Hsin-Yao Wang, Chia-Hsun Hsieh, Chiao-Ni Wen, Ying-Hao Wen, Chun-Hsien Chen, Jang-Jih Lu

**Affiliations:** 1 Department of Laboratory Medicine, Chang Gung Memorial Hospital at Linkou, Taoyuan City, Taiwan; 2 Division of Hematology-Oncology, Department of Internal Medicine, Chang Gung Memorial Hospital at Linkou and Chang Gung University, Taoyuan City, Taiwan; 3 Department of Information Management, Chang Gung University, Taoyuan City, Taiwan; 4 Department of Medical Biotechnology and Laboratory Science, Chang Gung University, Taoyuan City, Taiwan; Institute of Bioengineering and Nanotechnology, SINGAPORE

## Abstract

**Background:**

Analytic measurement of serum tumour markers is one of commonly used methods for cancer risk management in certain areas of the world (e.g. Taiwan). Recently, cancer screening based on multiple serum tumour markers has been frequently discussed. However, the risk–benefit outcomes appear to be unfavourable for patients because of the low sensitivity and specificity. In this study, cancer screening models based on multiple serum tumour markers were designed using machine learning methods, namely support vector machine (SVM), *k*-nearest neighbour (KNN), and logistic regression, to improve the screening performance for multiple cancers in a large asymptomatic population.

**Methods:**

AFP, CEA, CA19-9, CYFRA21-1, and SCC were determined for 20 696 eligible individuals. PSA was measured in men and CA15-3 and CA125 in women. A variable selection process was applied to select robust variables from these serum tumour markers to design cancer detection models. The sensitivity, specificity, positive predictive value (PPV), negative predictive value, area under the curve, and Youden index of the models based on single tumour markers, combined test, and machine learning methods were compared. Moreover, relative risk reduction, absolute risk reduction (ARR), and absolute risk increase (ARI) were evaluated.

**Results:**

To design cancer detection models using machine learning methods, CYFRA21-1 and SCC were selected for women, and all tumour markers were selected for men. SVM and KNN models significantly outperformed the single tumour markers and the combined test for men. All 3 studied machine learning methods outperformed single tumour markers and the combined test for women. For either men or women, the ARRs were between 0.003–0.008; the ARIs were between 0.119–0.306.

**Conclusion:**

Machine learning methods outperformed the combined test in analysing multiple tumour markers for cancer detection. However, cancer screening based solely on the application of multiple tumour markers remains unfavourable because of the inadequate PPV, ARR, and ARI, even when machine learning methods were incorporated into the analysis.

## Introduction

Several tools based on tumour markers have been developed for cancer risk management. However, each tumour marker test has been developed and validated for a specific category of anticipated cancer. In clinical practice, such tests are widely used in monitoring response to cancer treatment, but there is a lack of evidence supporting their use for screening multiple cancers. However, in certain areas of the world (e.g. Taiwan), people who fear of getting cancer often ask their clinicians to perform a combined set of common tumour marker tests to determine the likelihood of cancer development. In most cases, screening tests reveal no serologic sign of malignancy; however, some tests reveal suspicious results requiring further cancer surveying, with some patients were finally receiving a cancer diagnosis. In the literature, though lacking of evidence to be a screening tool, several studies have proposed that a panel of multiple serum tumour markers is probably more convenient and cost-effective for screening cancers in certain contexts because the cost of testing kits has decreased and the automated panel of testing has improved in recent years [1–8]. The true efficiency and the technique of statistical analysis of such panels remain unclear for populations who actively seek cancer screening through serologic testing.

Conventionally, according to the individual threshold value of tumour markers, the algorithm of the combined test would alarm patients with any one elevation of such tumour markers [1, 2, 7, 8]. However, the combined test has been proven to not significantly improve the discrimination ability of multiple tumour markers [6, 9]. The results of limited accuracy and evidence accordingly made the role of multiple tumour markers test insignificant and not recommended for routinely clinical use.

Recent studies have explored the application of machine learning methods in medical decision fields, including cancer risk prediction and aiding medical diagnosis [6, 9]. Several supervised machine learning methods, such as support vector machine (SVM), *k*-nearest neighbour (KNN), and logistic regression (LR) models, have received considerable attention in various medical applications over the past decades. Supervised machine learning methods can predict the class of an unknown case by generating a classification model from a set of training samples with a known class label. Multiple tumour markers subjected to the aforementioned machine learning methods have been proven to be superior to the combined test for diagnosing specific cancer types [6]. Several studies have also revealed that multiple variable analysis by using machine learning methods exhibits superior performance for cancer diagnosis or prognostic prediction compared with pathological studies [10, 11]. However, the effectiveness of applying machine learning methods to multiple tumour marker analysis for cancer screening has not yet been extensively established.

Therefore, we proposed that computational analysis techniques using the supervised learning methods SVM, KNN, and LR could probably develop classifiers for screening cancers by multiple tumour markers test. In this paper, we elucidate their efficiency and compare the performance of SVM, KNN, and LR models.

## Materials and Methods

### Patient Eligibility

This retrospective study was approved by the Ethics Committee of Chang Gung Memorial Hospital (IRB no. 104-4097B). Patient records were anonymised and de-identified prior to the analysis. We included 21 614 (9710 men and 11 904 women) apparently asymptomatic individuals who had at least once voluntarily undergone an out-of-pocket tumour marker panel test between March 2003 and December 2012 consecutively at the Linkou branch of Chang Gung Memorial Hospital [2]. We excluded 418 men and 500 women with previously diagnosed malignancies. All eligible individuals (9292 men and 11 404 women) had complete data on 6 tumour markers (AFP, CEA, CA19-9, CYFRA21-1, SCC, and PSA) for men and 7 tumour markers (AFP, CEA, CA19-9, CYFRA21-1, SCC, CA125, and CA15-3) for women [1, 2]. AFP, CEA, CA19-9, SCC, PSA, CA125, and CA15-3 were measured using commercially available kits (Abbott Diagnostics, Abbott Park, IL, USA). CYFRA21-1 was analytically determined with a commercially available kit (Roche Diagnostics Corp., Indianapolis, IN, USA). All assays of tumour markers met the requirements of the College of American Pathologists (CAP) Laboratory Accreditation Program, thus ensuring that the results were accurate and reproducible. Data were obtained from a cancer registry to determine whether each patient had received a new diagnosis of malignancy within 1 year of the tumour markers test. The data from the cancer registry revealed that of the 9292 men, 100 had received a diagnosis of malignancy within 1 year of the test. The cancer to noncancer ratio was 100:9192 for men. Similarly, of the 11 404 women, 87 had received a new diagnosis of malignancy within 1 year of the analytic measurements. The cancer to noncancer ratio was 87:11 317 for women.

Subsequently, a ratio of 2:1 (training to validation) was used to randomly allocate individuals to the training or validation set. All randomisations were performed using Matlab (MathWorks, Natick, MA, USA). For the men, 67 cases of newly diagnosed cancer and 6128 noncancer cases were randomised to the training set. Moreover, for the training set, random undersampling was applied [12–14] because of the extremely unbalanced data set used in this study. A cancer to noncancer ratio of 1:1 was adopted to randomise 67 individuals from the 6128 noncancer cases to the final training set. Consequently, the training set, which comprised 67 cases of newly diagnosed cancer and 67 noncancer cases, was used to train the machine learning models. For the women, 116 cases (58 newly diagnosed cancer cases and 58 noncancer cases) were randomised to the training set. In addition, one-third of all individuals were randomly allocated to the validation set to test the performance of the constructed models. The validation sets comprised 3097 cases (33 cases of newly diagnosed cancer and 3064 noncancer cases) for men and 3801 cases (29 cases of newly diagnosed cancer and 3772 noncancer cases) for women. The tumour types of occult cancer cases were also listed in the training and validation sets.

### Evaluation of Importance of Each Tumour Marker

To evaluate the importance of each tumour marker in the screening of cancers, a multivariate LR analysis of the tumour markers was performed for both sexes. Analyses were performed using SPSS (Version 20; SPSS Inc., Chicago, IL, USA). The continuous variables of all individuals were input to evaluate the significance. Results with *P* < .05 were considered statistically significant.

### Variable Selection on the Basis of the Youden Index

Using multiple variables in machine learning methods does not necessarily improve the prediction performance. The Youden index was used as a performance indicator for selecting the variables used in the classifier models in this study. The Youden index, which is among the most widely used performance indicators in biomedical studies, is calculated using the following formula: Youden index = Sensitivity + Specificity − 1. In this study, 6 and 7 tumour markers were analytically measured for the men and women, respectively. Therefore, 63 tumour marker combinations (**2**^**6**^ **− 1 = 63**) were evaluated for men and 127 (**2**^**7**^ **− 1 = 127**) for women. Each combination was evaluated using an LR classifier. To evaluate the performance of the classifiers, training and validation data sets were randomly constructed with a ratio of 2:1. The evaluation was repeated 100 times for each combination, and the Youden index values for each combination were averaged and compared. Only combinations with the highest averaged Youden index for each specific number of tumour markers were listed and compared. The appropriate combination of tumour markers for men and women were then used in the following experiments.

### Development of the SVM Models for Cancer Screening

In this study, we considered the binary classification problem. The discrimination ability of an SVM classifier is determined by generating a hyperplane in a high-dimensional space to discriminate the cancer group from the noncancer group. The SVM models used in this study were constructed using a Matlab version of the LIBSVM 3.20 software package, which is the most well-known and widely applied SVM software tool [15]. An effective SVM model was constructed using the procedures outlined in the manual by a previous study [16]. Briefly, the procedures mainly included 2 steps: (1) select an appropriate feature mapping kernel function such that the 2 groups might become linearly separable after mapping the samples into high-dimensional space, and (2) determine the parameters *c* (penalty for misclassification) and γ (function of the deviation of the radial basis function [RBF] kernel). In this study, the RBF kernel was selected. Previous research has proven that the RBF is superior to the linear kernel or sigmoid kernel in nonlinear classification problems such as cancer diagnosis [6]. This was confirmed in our preliminary trial. Subsequently, the values of *c* and γ were determined through an iterative grid search by 5-fold cross-validation in the training set, as detailed in previous studies [6, 16].

### Development of the KNN Algorithms for Cancer Screening

KNN is an instance-based algorithm used for classification. The KNN models used in this study were constructed using Matlab (MathWorks). In this study, the number of the nearest number was set to 7 according to our preliminary trial. For each case in the validation set, the Euclidean distances from the cases in the training set were calculated. The class categories of the 7 cases with Euclidean distances closest to the validation case were recorded. The class of the validation case was accordingly predicted on the basis of the major class categories of these 7 closest cases.

### Development of the LR Models for Cancer Screening

LR is a widely used and well-established methodology and is one of the most reliable classification methods for binary classification problems. The LR-based classifier was also constructed using Matlab (MathWorks). Training samples were used to determine the coefficients of each variable for the regression function, which was then used to further classify the validation cases. The probabilities of each validation case being classified as cancer and noncancer were set to *p* and *q* respectively, where *p* + *q* = 1. Subsequently, the odds (*p* divided by *q*) was used to predict the label of the validation cases. The cases were classified as cancer when the odds were one or more. Otherwise, the cases were classified as noncancer.

### Validation and Comparison of Various Models for Cancer Screening

The receiver operating characteristic (ROC) curve was used to evaluate the performance of the SVM-, KNN-, LR-based cancer screening models and the single tumour markers in panels. ROC curves for all machine learning methods and tumour markers were generated using SPSS (Version 20; SPSS Inc.). Furthermore, the area under the curve (AUC) was calculated to compare the discrimination abilities of machine learning methods and single tumour markers. Moreover, the performance of these machine learning methods was tested using the validation set. The performance of the combined test was also evaluated. The algorithm of the combined test was based on the threshold of each tumour marker. The thresholds of the tumour markers used in this study were 15 ng/mL for AFP, 5 ng/mL for CEA, 37 U/mL for CA19-9, 3.3 ng/mL for CYFRA21-1, 2.5 ng/mL for SCC, 4 ng/mL for PSA, 35 U/mL for CA125, and 30 U/mL for CA15-3. The sensitivity, specificity, positive predictive value (PPV), negative predictive value (NPV), and Youden index of the cancer screening models were calculated for the machine learning methods and the combined tests. The 95% confidence interval (CI) of the Youden indices of each method was calculated for further analysis, as detailed in previous studies [17, 18]. Moreover, for clinical consideration, the relative risk reduction (RRR), absolute risk reduction (ARR: cancer screened), and absolute risk increase (ARI: false diagnosis) were evaluated.

### Statistical Analyses

Data from the training and validation sets were analysed and are represented as the mean (median) ± standard deviation. An unpaired *t* test was used to compare the training and validation sets. The Fisher exact test was used to analyse the tumour types of occult cancer cases in the training and validation sets. Results with *P* < .05 were considered statistically significant. To evaluate the importance of each tumour marker, the standard error (SE) of the coefficients and the mean and 95% CI of odds ratios were calculated for each tumour marker. One-way analysis of variance (ANOVA) with a statistical significance level of 0.05 was used to examine the effects of the different tumour markers combinations on the Youden index values, AUC values of various machine learning methods and single tumour markers, and Youden index values of machine learning methods and the combined tests. The Tukey honestly significant difference post hoc test was used to determine the differences when the null hypothesis of ANOVA was rejected. Results with *P* < .05 or < .01 were labelled separately. All statistical analyses were performed using SPSS (Version 20; SPSS Inc.).

## Results

### General Patient Characteristics

Of 21 614 individuals, 918 individuals were excluded; the remaining 20 696 eligible individuals were included in this study. In the step of random under-sampling, the training sets comprised 134 and 116 cases for men and women, respectively. Moreover, 3097 and 3801 cases for men and women, respectively, were randomly allocated to the validation set at a ratio of 2:1 (training to validation), as shown in Table 1. In the descriptive analysis, variables including age and the tumour markers were compared between the training and validation sets. For the men and women, the average age of the cases in the validation set was significantly less than those in the training set. In addition, for the men, the average CYFRA21-1 and CA19-9 values in the validation set were significantly lower than those in the training set. For the women, only the average CYFRA21-1 value was significantly lower in the validation set. The distribution of other tumour markers was similar between the two sets. For the men, the major tumour types of occult cancer in the training set were prostate (20.90%), colorectal (13.43%), lung (10.45%), liver (10.45%), and head and neck (7.46%) cancers; the major tumour types in the validation set were lung (18.18%), head and neck (15.15%), urinary (15.15%), prostate (9.09%), and hematopoietic and lymphoid (9.09%) cancers. For the women, the major tumour types of occult cancer in the training set were breast (25.86%), gynecologic (22.41%), thyroid (12.07%), lung (8.62%), and gastric (6.90%) cancers; the major tumour types in the validation set were breast (34.48%), gynecologic (24.41%), thyroid (20.69%), lung (6.90%), and colorectal (6.90%) cancers, as shown in Table 2. No significant differences were observed in the distribution of the tumour types of occult cancer between the training and validation sets for both men and women.

**Table 1:** Clinicopathological Information for the Training and Validation Sets.

Variable	Training Set	Validation Set	p-value
**Male**			
No. of patients	134	3097	-
Age (yr)	57. 19 (57) ± 14.05	50.59 (50) ± 12.33	<0.001*
AFP (ng/mL)	2176.31 (3.44) ± 25124.34	77.83 (3.24) ± 4111.53	0.336
CEA (ng/mL)	6.40 (2.3) ± 26.45	2.23 (1.84) ± 2.09	0.07
CA19-9 (U/mL)	15.72 (7.23) ± 32.25	8.38 (5.00) ± 12.01	0.01*
CYFRA21-1 (ng/mL)	2.07 (1.80) ± 1.15	1.68 (1.47) ± 0.98	<0.001*
SCC (ng/mL)	0.71 (0.40) ± 0.91	0.62 (0.40) ± 0.48	0.243
PSA (ng/ml)	15.06 (1.04) ± 140.91	1.34 (0.80) ± 2.24	0.262
**Female**			
No. of patients	116	3801	-
Age (yr)	51.16 (51) ± 12.44	48.25 (48) ± 11.57	0.014*
AFP (ng/ml)	4.06 (3.09) ± 6.59	3.54 (2.96) ± 4.07	0.405
CEA (ng/ml)	5.88 (1.47) ± 28.70	1.85 (1.28) ± 17.56	0.136
CA19-9 (U/ml)	11.58 (6.72) ± 13.09	16.15 (6.43) ± 302.69	0.367
CYFRA21-1 (ng/ml)	1.92 (1.46) ± 2.21	1.40 (1.23) ± 0.84	0.014*
SCC (ng/ml)	0.60 (0.40) ± 0.89	0.51 (0.30) ± 0.59	0.325
CA125 (U/ml)	17.45 (9.875) ± 23.79	14.54 (10) ± 24.62	0.197
CA15-3 (U/ml)	10.36 (8.65) ± 5.07	9.75 (8.50) ± 4.77	0.206

Data are presented as mean (median) ± standard deviation. Significant differences are denoted by * (*P* < .05).

**Table 2:** Occult Cancer Tumour Types for the Training and Validation sets.

Tumour Type	Training Set		Validation Set		
**Male**	no. of tumours/total no.	(%)	no. of tumours/total no.	(%)	p-value
Lung	7/67	10.45%	6/33	18.18%	0.35
Liver	7/67	10.45%	2/33	6.06%	0.71
Colorectal	9/67	13.43%	1/33	3.03%	0.16
Prostate	14/67	20.90%	3/33	9.09%	0.17
Thyroid	4/67	5.97%	2/33	6.06%	1.00
Gastric	4/67	5.97%	1/33	3.03%	1.00
Pancreas	3/67	4.48%	1/33	3.03%	1.00
Bile duct	1/67	1.49%	1/33	3.03%	1.00
Head & Neck	5/67	7.46%	5/33	15.15%	0.29
Urinary	3/67	4.48%	5/33	15.15%	0.11
Hematopoietic & lymphoid	3/67	4.48%	3/33	9.09%	0.39
Skin	4/67	5.97%	1/33	3.03%	1.00
CNS	1/67	1.49%	0/33	0%	1.00
Thymus	0/67	0%	1/33	3.03%	0.33
Unknown primary	2/67	2.99%	1/33	3.03%	1.00
**Female**					
Lung	5/58	8.62%	2/29	6.90%	1.00
Liver	1/58	1.72%	1/29	3.45%	1.00
Colorectal	3/58	5.17%	2/29	6.90%	1.00
Breast	15/58	25.86%	10/29	34.48%	0.46
Gynecologic	13/58	22.41%	7/29	24.14%	1.00
Thyroid	7/58	12.07%	6/29	20.69%	0.34
Gastric	4/58	6.90%	0/29	0.00%	0.30
Pancreas	1/58	1.72%	0/29	0.00%	1.00
Bile duct	1/58	1.72%	0/29	0.00%	1.00
Head & Neck	2/58	3.45%	0/29	0.00%	0.55
Urinary	2/58	3.45%	1/29	3.45%	1.00
Hematopoietic & lymphoid	1/58	1.72%	0/29	0.00%	1.00
Skin	1/58	1.72%	0/29	0.00%	1.00
Unknown primary	2/58	3.45%	0/29	0.00%	0.55

### Variable Significance for Men and Women

Multivariate LR analysis was performed to evaluate the significance of each variable. Tables 3 and 4 present the coefficients of the variables in the regression equation and the significance of each variable. For the men, 4 tumour markers (CEA, CA19-9, CYFRA21-1, and PSA) were significantly associated with the rate of cancer diagnosis, as shown in Table 3. For the women, 2 tumour markers (CYFRA21-1 and CA15-3) were significantly associated with the rate of newly diagnosed cancers in multivariate analysis, as shown in Table 4.

**Table 3:** Results of the Multivariate LR Analysis (Male).

Variable	Coefficient	SE	Odds Ratio	95% CI	p-value
AFP	.000	.006	1.000	.989–1.011	0.987
CEA	.185	.029	1.203	1.137–1.274	<0.001*
CA19-9	.005	.002	1.005	1.001–1.009	0.020*
CYFRA21-1	.409	.063	1.505	1.331–1.701	<0.001*
SCC	-.276	.200	.759	.512–1.124	0.169
PSA	.073	.014	1.076	1.047–1.106	<0.001*
Constant	-5.934	.221	.003		<0.001*

Significant differences are denoted by * (*P* < .05). (SE: standard error; CI: confidence interval)

**Table 4:** Results of the Multivariate LR Analysis (Female).

Variable	Coefficient	SE	Odds Ratio	95% CI	p-value
AFP	.005	.016	1.005	.973–1.038	0.744
CEA	-.003	.003	.997	.991–1.003	0.282
CA199	.002	.001	1.002	.999–1.004	0.186
CYFRA21-1	.280	.050	1.323	1.199–1.460	<0.001*
SCC	.048	.071	1.050	.914–1.206	0.493
CA125	-.001	.001	.999	0.997–1.001	0.502
CA15-3	.038	.017	1.039	1.006–1.074	0.020*
Constant	-5.799	.235	.003		<0.001*

Significant differences are denoted by * (*P* < .05). (SE: standard error; CI: confidence interval)

### Variable Selection for Men and Women

**Fig 1:**
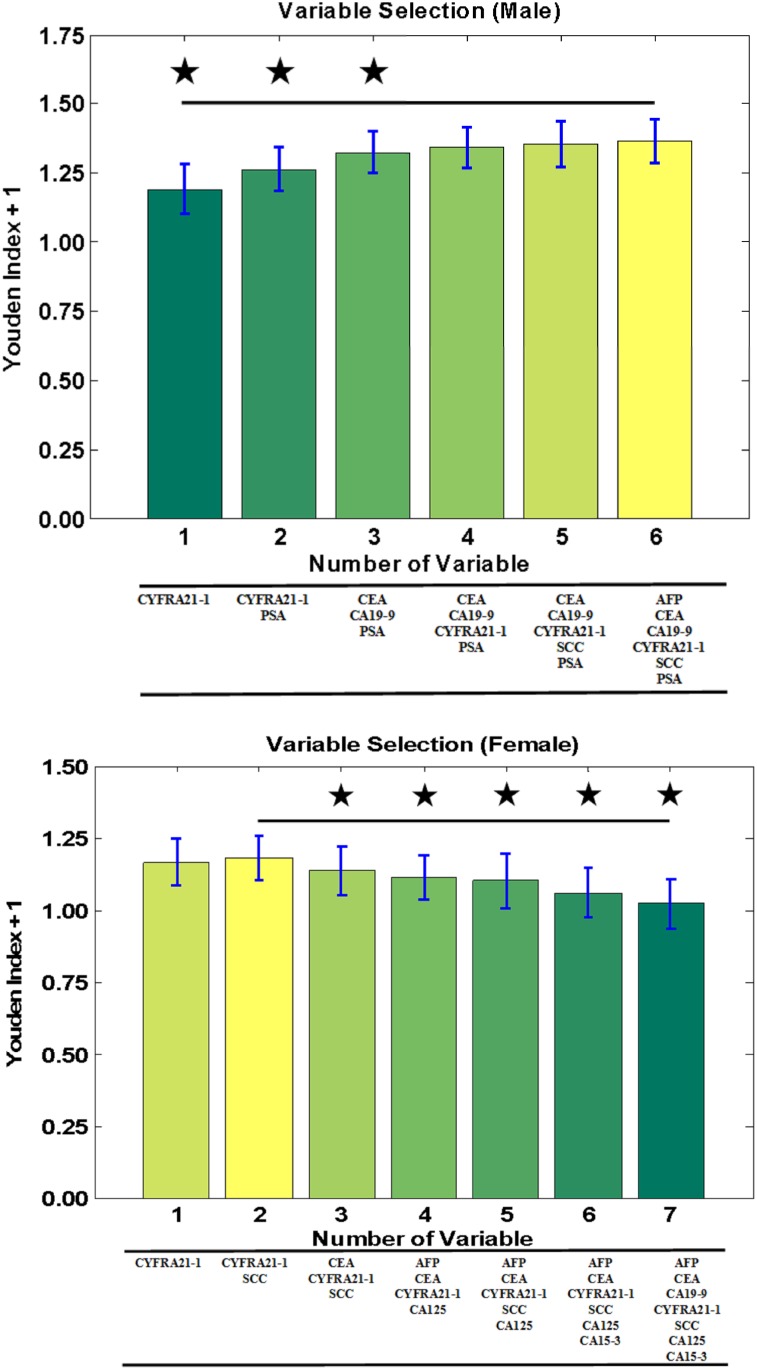
**(a) Variable Selection (Male).** Evaluation of Youden index values (expressed as Youden index + 1) under various tumour markers combinations are displayed as the mean ± the standard deviation for each combination (as indicated). Significant differences are denoted by ★ (*P* < .05). **(b) Variable Selection (Female).** Evaluation of Youden index values (expressed as Youden index + 1) under different combinations of tumour markers are displayed as the mean ± the standard deviation for each combination (as indicated). Significant differences are denoted by ★ (*P* < .05).

Six (AFP, CEA, CA19-9, CYFRA21-1, SCC, and PSA) and 7 (AFP, CEA, CA19-9, CYFRA21-1, SCC, CA125, and CA15-3) tumour markers were measured for the men and women, respectively. Accordingly, 63 combinations of tumour markers for men and 127 for women were evaluated using the Youden index to select an appropriate combination of variables for constructing effective cancer classification models with the highest sensitivity and specificity. As shown in Fig 1(a), the combination of AFP, CEA, CA19-9, CYFRA21-1, SCC, and PSA attained the highest Youden index values for the men. By contrast, for the women, the combination of CYFRA21-1 and SCC exhibited significantly higher Youden index values compared with the other combinations, as shown in Fig 1(b). Consequently, to construct machine learning models for screening of cancers, all tested tumour markers were selected for the men, but only CYFRA21-1 and SCC were selected for the women.

### Performance for General Cancers Screening

**Fig 2:**
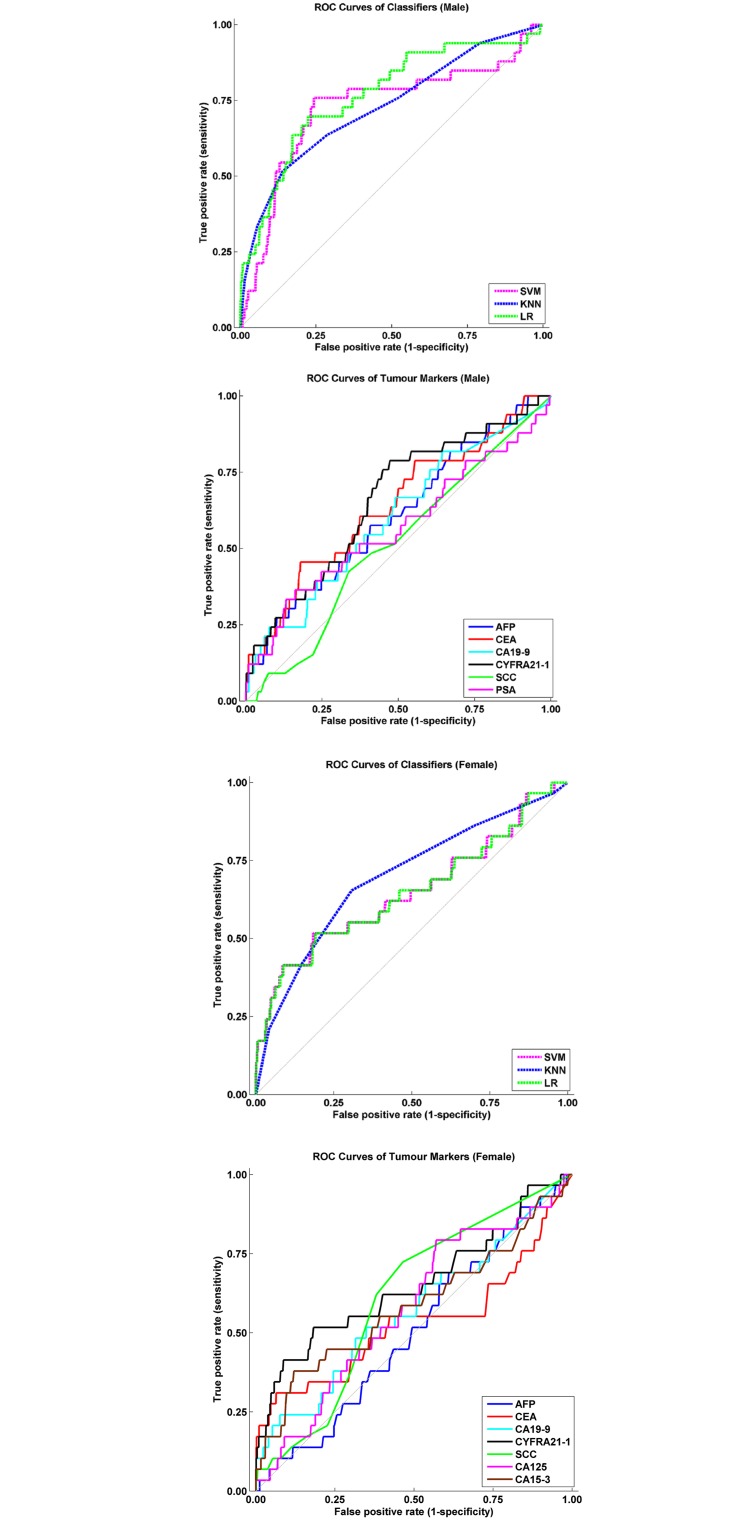
(a) ROC Curves of the Various Machine Learning Models for Cancer Screening (Male). (b) ROC Curves of the Various Tumour Markers for Cancer Screening (Male). (c) ROC Curves of the Various Machine Learning Models for Cancer Screening (Female). (d) ROC Curves of the Various Tumour Markers for Cancer Screening (Female).

ROC curves and AUC values were used to assess the performance of the various machine learning methods and each tumour marker for cancer surveying (Fig 2, Tables 5 and 6). For the men, the performance of the machine learning methods in the analysis of the multiple tumour markers was generally superior to those of the single tumour markers based on the AUC values, as shown in Fig 2(a) and 2(b) and Table 5. The AUC values of the SVM, KNN, and LR models were significantly higher than those of AFP (*P* < .01), CEA (*P* < .01), CA19-9 (*P* < .01), CYFRA21-1 (*P* < .01), SCC (*P* < .01), and PSA (*P* < .01). For the women, the various machine learning methods used in the analysis of CYFRA21-1 and SCC also outperformed the single tumour markers (*P* < .01), except for CYFRA21-1, as shown in Fig 2(c) and 2(d) and Table 6.

**Table 5:** AUC Values of Various Classifiers and Tumour Markers for Cancer Screening (Male).

Classifier/Tumour marker	Area under the curve	95% CI
SVM	.726	.621-.831
KNN	.727	.630-.825
LR	.766	.676-.856
CYFRA21-1	.657	.562-.752
CEA	.639	.538-.741
AFP	.607	.507-.706
CA19-9	.599	.498-.701
PSA	.568	.454-.682
SCC	.514	.418-.609

CI: confidence interval

**Table 6:** AUC Values of the Various Classifiers and Tumour Markers for Cancer Screening (Female).

Classifier/Tumour marker	Area under the curve	95% CI
SVM	.650	.529-.771
KNN	.699	.594-.804
LR	.649	.528-.770
CYFRA21-1	.651	.530-.771
SCC	.610	.518-.703
CA15-3	.583	.459-.708
CA125	.576	.472-.679
CA19-9	.572	.456-.688
CEA	.531	.394-.668
AFP	.504	.403-.605

CI: confidence interval

For the men (Table 7), the SVM model attained the highest sensitivity (0.758), whereas KNN algorithm attained the highest specificity (0.862) and PPV (0.039). All methods attained high NPVs (all higher than 0.994). Among the machine learning methods, the SVM model and the KNN algorithm, but not the LR model, attained significantly higher Youden index values than the combined test (*P* < .01). Moreover, the SVM model attained higher Youden index values compared with the KNN and LR models and the combined test (*P* < .01). By contrast, for the women (Table 8), the KNN algorithm attained the highest sensitivity (0.655), whereas the combined test attained the highest specificity (0.880) and PPV (0.022). Moreover, all the methods attained high NPVs (all higher than 0.994). The SVM, KNN, and LR models attained significantly higher Youden index values (*P* < .01) compared with the combined tests. Moreover, the SVM model outperformed both the KNN and LR model, as indicated by the significantly higher Youden index (*P* < .01). Additionally, the ARR, RRR, and ARI are reported in Tables 9 and 10. For the men, the ARRs ranged from 0.005 to 0.008, with the SVM model attaining the highest ARR (0.008). The number needed to treat (NNT) was calculated from the ARR. The NNTs for the males ranged from 125 to 200. The ARIs ranged from 0.137 to 0.241, where resulted in numbers needed to harm (NNHs) ranging from 4 to 7. For the women, the ARRs ranged from 0.003 to 0.005, with the KNN model attaining the highest ARR (0.005). Consequently, the NNTs for the females ranged from 200 to 333. Besides, the ARIs ranged from 0.119 to 0.306. The calculated NNHs ranged from 3 to 8.

**Table 7:** Performance of the Various Methods for Cancer Screening (Male).

	Sensitivity (95% CI)	Specificity (95% CI)	PPV (95% CI)	NPV (95% CI)	Youden Index (95% CI)
**SVM**	.758 (.612-.904)	.757 (.742-.772)	.032 (.020-.044)	.997 (.994-.999)	.514 (.403-.626) **
**KNN**	.515 (.345-.686)	.862 (.850-.874)	.039 (.020-.057)	.994 (.991-.997)	.377 (.230-.524) **
**LR**	.485 (.315-.656)	.859 (.847-.871)	.036 (.019-.053)	.994 (.991-.997)	.344 (.197-.490)
**Combined Test of 6 Tumour Markers**	.515 (.345-.686)	.851 (.838-.864)	.036 (.019-.052)	.994 (.991-.997)	.366 (.220-.511)

The Youden index values of the SVM, KNN, and LR models were compared with the combined test. Significantly higher differences are denoted by ** (*P* < .01).

**Table 8:** Performance of the Various Methods for Cancer Screening (Female).

	Sensitivity (95% CI)	Specificity (95% CI)	PPV (95% CI)	NPV (95% CI)	Youden Index (95% CI)
**SVM**	.517 (.335-.699)	.816 (.804-.828)	.016 (.007-.025)	.996 (.994-.998)	.347 (.198-.500) **
**KNN**	.655 (.482-.828)	.691 (.676-.706)	.021 (.013-.029)	.995 (.993-.998)	.333 (.213-.453) **
**LR**	.517 (.335-.699)	.758 (.744-.772)	.016 (.008-.024)	.995 (.992-.998)	.275 (.137-.414) **
**Combined Test of 7 Tumour Markers**	.345 (.172-.518)	.880 (.870-.890)	.022 (.009-.035)	.994 (.991-.997)	.225 (.073-.377)

The Youden index values of the SVM, KNN, and LR models were compared with the combined test. Significantly higher differences are denoted by ** (*P* < .01).

**Table 9:** RRR, ARR, and ARI of the Various Machine Learning Methods and the Combined Test (Male).

	RRR (95% CI)	ARR (95% CI)	ARI (95% CI)
**SVM**	.758 (.623-.845)	.008 (.004-.012)	.241 (.226-.256)
**KNN**	.515 (.317-.655)	.006 (.003-.008)	.137 (.124-.149)
**LR**	.485 (.280-.632)	.005 (.003-.008)	.140 (.128-.152)
**Combined Test of 6 Tumor Markers**	.515 (.317-.655)	.006 (.003-.008)	.148 (.135-.160)

**Table 10:** RRR, ARR, and ARI of the Various Machine Learning Methods and the Combined Test (Female).

	RRR (95% CI)	ARR (95% CI)	ARI (95% CI)
**SVM**	.517 (.303-.665)	.004 (.002-.006)	.183 (.171-.195)
**KNN**	.655 (.478-.772)	.005 (.003-.007)	.306 (.291-.321)
**LR**	.517 (.303-.665)	.004 (.002-.006)	.240 (.226-.254)
**Combined Test of 7 Tumor Markers**	.345 (.086-.531)	.003 (.001-.005)	.119 (.109-.129)

## Discussion

Screening of cancers has received considerable attention in developed and developing countries owing to the heavy economic and quality-of-life burden caused by cancers. Although testing for multiple tumour markers in cancer screening lacks sufficient evidence for evaluating its effectiveness and it is associated with adverse risk–benefit outcomes, it is widely used in certain areas (e.g. Taiwan). The present study focused on determining whether machine learning methods can improve the discrimination ability of multiple tumour markers for cancer screening. In Taiwan, more than 96% of the population is covered by the National Health Insurance (NHI) program [19]. Patients covered by the NHI program can seek medical help for their symptoms without paying additional money. In this study, 20,696 individuals who had at least once undergone an out-of-pocket health check-up were considered apparently asymptomatic [1, 2]. Those who had previously received a cancer diagnosis before undergoing the tumour marker test were excluded from the analysis. Given the high case number and the relatively long study period (approximately 10 y), the cases included in this study are representative of the health check-up population in Taiwan.

Because of the extremely unbalanced data set used in this study, in which the noncancer cases outnumbered the cancer cases by a ratio of approximately 100:1, using an appropriate method for partitioning the data set into the training and validation sets was crucial. Given that most machine learning methods are generally developed for balanced data sets, improved results might be obtained when pre-analysis processing is performed for original data [12–14]. In this study, random under-sampling was used to create a balanced training set, in which the number of cancer cases was identical to that of noncancer cases. By contrast, the ratio of cancer to noncancer cases was unchanged for the validation set. Therefore, the proportion of cancer cases in the validation set was identical to that in the original cohort. This arrangement enabled calculating the PPV and NPV of each classifier. Both the PPV and NPV values are crucial information in making clinical management decisions.

The distribution of a few variables differed between the training and validation sets, as shown in Table 1. Specifically, for the men, the average age, CA19-9, and CYFRA21-1 were significantly lower in the validation set. For the women, the average age and CYFRA21-1 were significant lower in the validation set. Generally, in clinical practice, correctly interpreting tumour marker results is relatively difficult for physicians when patients are younger and the analytical levels of tumour markers are lower. Despite these distribution differences between the training and validation sets, these data may ensure that the improved discrimination ability was not because of the relatively easy validation set.

Appropriate variable selection can reduce the number of tumour markers in cancer screening models constructed using machine learning methods. In addition, variable reduction would result in less calculation, leading to less computation-intensive models. For variable selection, multivariate LR analysis and the Youden index were used and compared in this study. Variables significantly associated with the cancer screening outcomes would be selected as appropriate variables in multivariate LR analysis [20]. In this study, multivariate LR analysis results revealed that CEA, CA19-9, CYFRA21-1, and PSA and CYFRA21-1 and CA15-3 were significantly associated with the prediction outcomes of cancer screening for the men (Table 3) and women (Table 4). However, the combination of all 6 tumour markers attained the highest Youden index values for the men (Fig 1(a)). Moreover, the combination of CYFRA21-1 and SCC attained the highest Youden index values for the women (Fig 1(b)). Although discordance was observed between these 2 methods, the results of the Youden index were adopted. The Youden index is simple to calculate and exhibits a linear relationship with the AUC [21]. In addition, both the sensitivity and specificity of a cancer screening classifier model can be optimised as much as possible by using the Youden index.

The performance of the machine learning methods evaluated in this study was generally higher than that of all single tumour markers (Tables 5 and 6) and the combined test for cancer screening (Tables 7 and 8). The SVM, KNN, and LR models are all superior classifiers whose abilities to support medical decision have been widely studied [9, 10, 20, 22, 23]. It is reasonable to expect that the performance of multiple tumour markers combined with machine learning methods would be higher than that of the single tumour markers for cancer screening, because multiple variables may provide additional information. Moreover, single tumour markers are not recommended as a tool for cancer screening or diagnosis (3). A single threshold is determined for each tumour marker on the basis of statistical analysis. However, a single threshold value is difficult to determine when all tumour markers are combined together. Consequently, the discrimination ability of the combined test might be compromised. By contrast, machine learning methods learn from the distribution pattern of all variables for a specific classification problem. Consequently, the performance of machine learning methods might be optimised as much as possible; thus, these methods are superior to the combined test, in which the threshold value for each tumour marker must be determined independently.

By contrast, for the women, the performance of the machine learning methods, single tumour markers, and combined test was not as high as those for the men. The underlying reasons might be complicated. First, the menstrual cycle may cause fluctuations in multiple endocrines. Studies have described the effect of endocrine levels on the tumour marker levels [24–27]. The level of the well-known tumour marker CA125 is mainly elevated during the menstrual cycle. Moreover, AFP is reported to be elevated during some specific period or situation of pregnancy [25]. Furthermore, diseases specifically affecting women, such as benign uterus diseases, pelvis inflammatory diseases, or benign breast tissue diseases, are involved in the activation of epithelial cells [26, 27]. All these diseases may cause elevation of epithelium-associated tumour markers in noncancerous diseases. In this situation, incorporating machine learning methods into analysis of multiple tumour markers still yielded higher performance compared with the combined test.

The NPVs of all the machine learning methods and the combined test were high (Tables 7 and 8). Consequently, either method is appropriate for excluding the risk of cancer when the prediction is negative. However, the PPVs of all methods were low (1.6%–3.9%) (Tables 7 and 8). For rare diseases, which inherently have low prevalence and incidence, mathematically low PPVs might be obtained even when the classifier has high sensitivity and specificity [28]. In the included cohort, cancer prevalence was low (1.08% and 0.76% in men and women, respectively). Consequently, a low PPV level would be almost inevitably obtained for the cancer screening models in this study. High PPV performance could be obtained through simulations with higher prevalence. Moreover, PPV performance is mathematically associated with sensitivity, specificity, and disease prevalence. If the prevalence of cancers increased to 10% in men, the PPVs would be elevated to 0.257, 0.293, 0.277, and 0.278 for the SVM, KNN, LR, and combined test, respectively. Similarly, PPVs would be elevated to 0.238, 0.191, 0.192, and 0.242 for SVM, KNN, LR, and combined test in female population.

Several other studies have applied machine learning methods to analyse data from microarrays to screen or diagnose cancers [29, 30]. These studies have generally demonstrated substantially high discrimination ability for malignancy. However, the high cost of microarrays might prevent their wide application for screening cancers in the general population. A practical and ideal screening tool should exhibit adequate sensitivity, specificity, and cost-effectiveness. In this study, all analytical measurements were performed in a CAP-accredited laboratory, and the test results are accurate and reproducible. Application of mmachine learning methods to analysis of multiple tumour markers for cancer screening was studied. The results showed that the performance of the machine learning methods was generally higher than that of the combined test for cancer screening. Nevertheless, this study has some limitations. First, analytic measurement of multiple tumour markers for cancer screening has not yet been considered as an evidence-based practice. Additionally, given the retrospective nature of this study, few individuals with ongoing but unconfirmed examinations would be misclassified into the noncancer class. Moreover, all individuals in this study spent additional money to undergo out-of-pocket health examinations. They were assumed to have a relatively higher financial status. It would be questionable to generalise the results derived from such a sample to the general population in Taiwan. Moreover, although cases were collected over approximately 10 years in this study, every category of cancer may have not been covered owing to the paucity of cases with occult cancer (Table 2). To train a reliable model, more data distribution patterns of various cancer types must be included. Overall, analysis of the multiple tumour markers by using various machine learning methods improved the performance of the multiple tumour markers for identifying occult cancers in the apparently asymptomatic population.

For clinical consideration, however, the ARRs and ARIs (Tables 9 and 10) in this study indicate that analytical measurement of multiple tumour markers for cancer screening is not favourable. On the basis of the ARRs and ARIs, 1 in 125–200 males were helped (cancer screened), whereas 1 in 4–7 males were harmed (false diagnosis). Similarly, 1 in 200–333 females were helped, whereas 1 in 3–8 females were harmed. Although the machine learning methods attained higher ARRs than the combined tests, their ARRs and ARIs were inadequate for clinical application for either men or women. It means that machine learning methods could mine the maximal values out of multiple tumour markers. Nevertheless, routine use of multiple tumour markers for cancer screening is not recommended because the clinical indicators were still not improved adequately by the machine learning methods.

## Conclusion

The machine learning methods investigated in this study outperformed the combined tests in the analysis of multiple tumour markers for discriminating cancer cases from noncancer cases. However, cancer screening based solely on the use of multiple tumour markers remains unfavourable because of the inadequate PPVs, ARRs, and ARIs, even after incorporating the machine learning methods into the analysis.
